# Exceedingly rare incidence of a double inferior vena cava (IVC) with azygos continuation of left IVC

**DOI:** 10.1002/ccr3.8981

**Published:** 2024-05-23

**Authors:** Adeleh Dadkhah, Saeed Akbarzadeh Pasha, Mohammad Amin Borjian

**Affiliations:** ^1^ Department of Radiology, School of Medicine Iran University of Medical Sciences Tehran Iran

**Keywords:** azygos continuation of left IVC, case report, double inferior vena cava, left renal vein, left‐side inferior vena cava

## Abstract

**Key Clinical Message:**

Because of the complex embryonic origin of the abdominal venous structures, IVC and azygous systems can show numerous and even previously unreported anatomical variations and anomalies. Also, evaluating major vascular structures should not be dismissed in non‐contrast‐enhanced CT as it can provide valuable information about these structures.

**Abstract:**

Double IVC is a rare occurrence of IVC anatomical variations and congenital anomalies. Herein, we discuss a case of a very rare type of double IVC that has not been reported in the literature before. A non‐contrast‐enhanced CT study was performed for a 34‐year‐old patient who visited our ER to evaluate for urolithiasis, during which two IVCs were noted. Each renal vein joined the ipsilateral IVC at a perpendicular angle. Unusually, the right IVC was formed from the confluence of both left and right common iliac veins (CIV), and the left IVC—Instead of crossing the midline at the renal veins level and reuniting the right IVC—cranially contributed to the azygos vein formation and caudally joined the left CIV. Also, there were some small communicating veins between the two IVCs and the left gonadal vein was slightly dilated before suggesting a reflux from the left renal vein (LRV). A complimentary doppler ultrasound exam confirmed the diagnosis and revealed a left‐side varicocele. Although rare cases of hemiazygos continuation and interiliac connections of left‐side IVC in the cases of double‐IVC have been reported previously, a complete confluence of CIVs is rare. The main differential diagnosis is retro‐aortic left renal vein (RLRV) type IV which seems to have an oblique course. Radiologists and surgeons should expect previously unreported variations in the vena cava system. Furthermore, reviewing the main abdominal vasculature should not be dismissed in non‐contrast CT exams.

## Graphical abstract

**The operator assisted 3D reconstruction of the main retroperitoneal vasculature:** the Aorta is shown in red; the right renal vein, right Inferior Vena Cava (IVC), and common iliac veins are depicted in dark blue; the left renal vein, left IVC, azygos vein, and accessory hemiazygos vein are illustrated in cyan; and the gonadal veins are represented in green. The left IVC exhibits multiple connections to the right IVC and the left Common Iliac Vein (CIV) caudally. It follows a vertical path and intersects with the left renal vein at a right angle. The superior segment of the left IVC, (arrow) does not merge with the right IVC after crossing the midline above the confluence of the left renal vein. Instead, it contributes to the formation of the azygos vein at the T12 vertebral level.

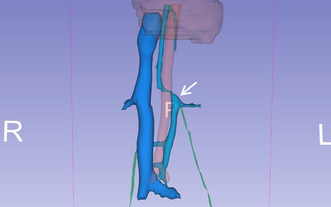

## 1 INTRODUCTION

According to various studies, inferior vena cava (IVC) can show different anatomical variations and anomalies with a reported prevalence of 0.5%–4% in the general population.^1^, ^2^ Some of these variations, such as double IVC and retro‐aortic left renal vein (RLRV), are familiar to radiologists and surgeons worldwide; some of them like the absence of infrarenal IVC are rarely reported in the literature.^3^, ^4^ These variations, alongside their embryological origins, their connections to the azygos system, and their clinical significance are well categorized in several sources; especially in the past two decades that computerized tomography (CT) scan has become a mainstream and readily available diagnostic tool. Renal veins, more specifically on the left side, may be affected by many of these anatomical variations.^1^, ^4^, ^5^ This report presents a very rare drainage course of the LRV into a double IVC in a patient who had undergone a CT scan for an unrelated reason.

## 2 CASE PRESENTATION

A 34‐year‐old male with an unremarkable past medical history, presented with acute left flank pain at our center. With the suspected cause of urolithiasis, the patient underwent a non‐contrast‐enhanced CT (NCECT) scan. The emergency physician detected a stone measuring 10 mm in the middle segment of the left ureter. The patient was subsequently discharged after pain management and scheduled for an outpatient follow‐up in the urology clinic.

**FIGURE 1:**
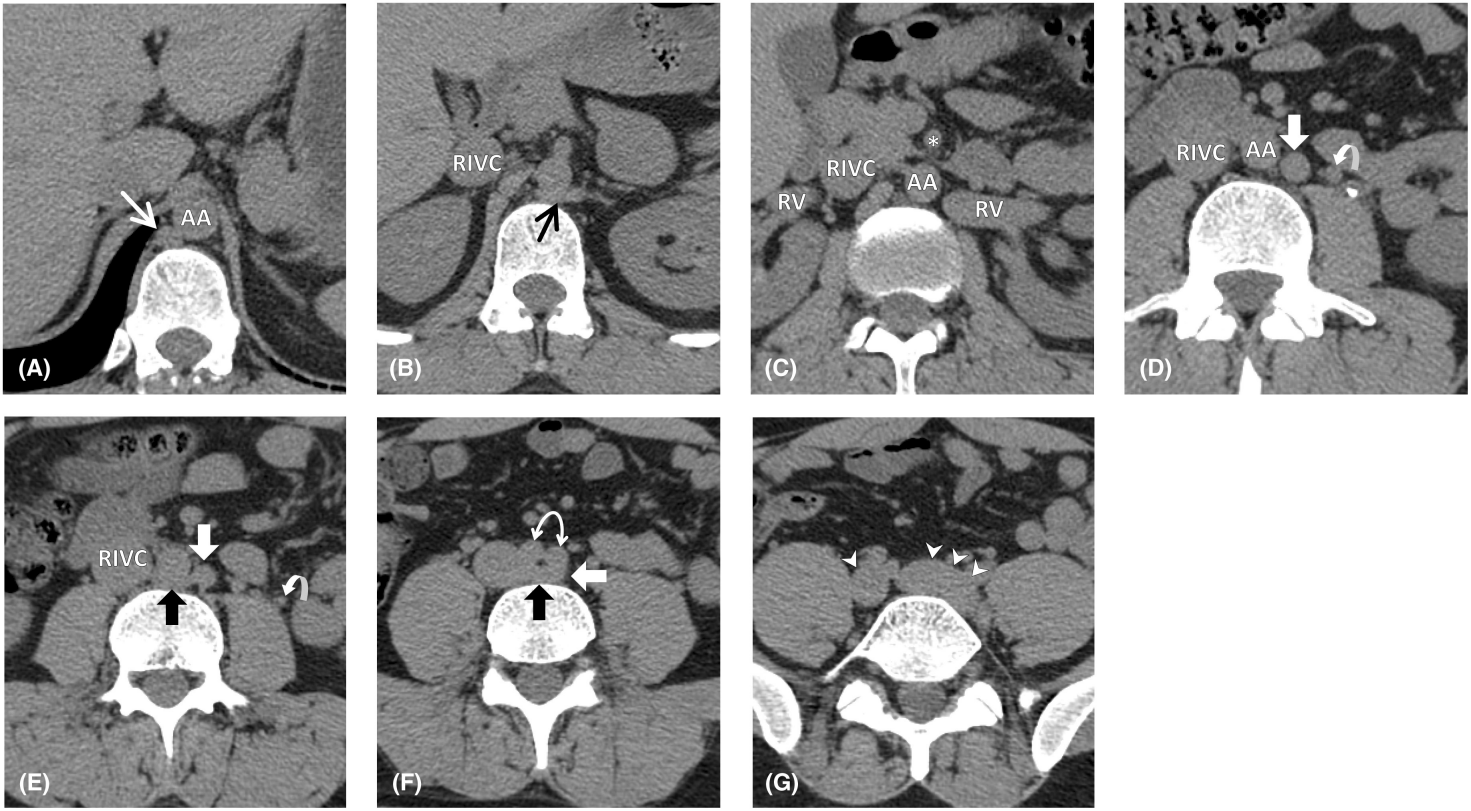
Axial planes of non‐contrast‐enhanced Abdominopelvic CT at different levels. Images were cropped for better visualization of retroperitoneal vascular structures. from cranial to caudal: (A) at the level of T12 vertebra. Azygos vein (white arrow) and aorta (AA) (B) at the level of L1 vertebra. There is a communicating horizontal venous structure (black arrow) crossing the midline between aorta and vertebral body, connecting the left IVC to the azygos vein in image A. Right IVC (RIVC) (C) at the level of L1‐L2 intervertebral disc through the renal veins (RV). The left renal vein does not cross anterior to the abdominal aorta and superior mesenteric artery (*) as it's expected to. (D) at the level of L3 vertebra. There is an additional venous structure (white blocked arrow) left to the abdominal aorta which was identified as left IVC. A slightly prominent left gonadal vein (curved arrow) and ureter stone can also be seen. (E) at the level of L3 vertebra just below image D. On careful evaluation, small communications (black blocked arrow) between left and right IVCs are visible crossing anterior to the vertebral bodies. (F) at the level of L4 vertebra right after abdominal aorta bifurcation into common iliac arteries (bidirectional curved arrow) (G) at the level of L5 vertebra through common iliac veins (arrowheads). The left IVC has already joined the left CIV.

**FIGURE 2:**
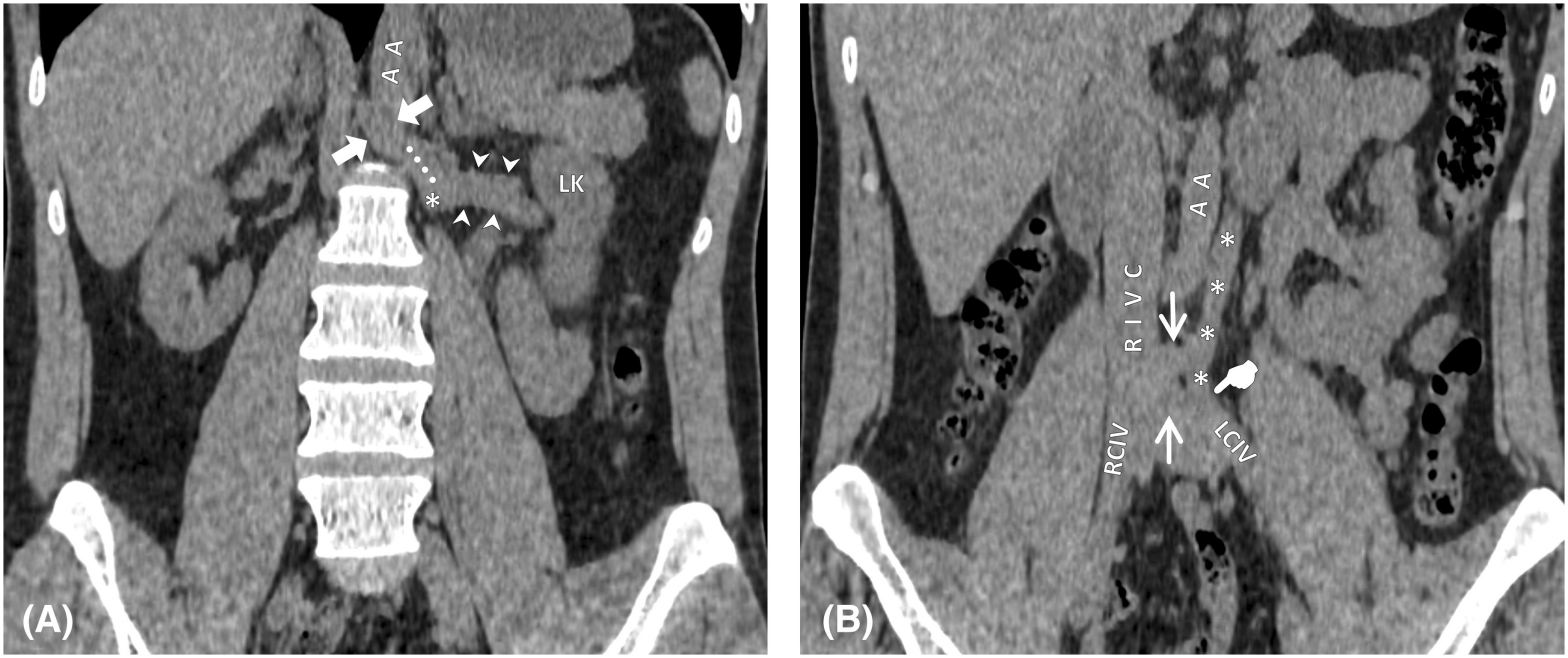
Coronal reconstructions of non‐contrast‐enhanced Abdominopelvic CT at different levels. Images were cropped for better visualization of retroperitoneal vascular structures. (A) coronal plane through anterior aspects of lumbar vertebrae. Left renal vein (between arrowheads) connects to the left IVC (*) at a right angle. There is a superior 8 mm segment (dotted line) of left IVC above the confluence of the LRV that contributes to azygos vein formation via a horizontal communicating vein (between blocked arrows) that crosses anterior to the L1 vertebra. Posterior aspects of left kidney (LK) and aorta (AA) are also shown. (B) Another coronal plane 1 cm anterior to image A, demonstrating most of the right IVC (RIVC), left IVC route (****), their communications (arrows), and left IVC's connection to the left CIV.

**FIGURE 3:**
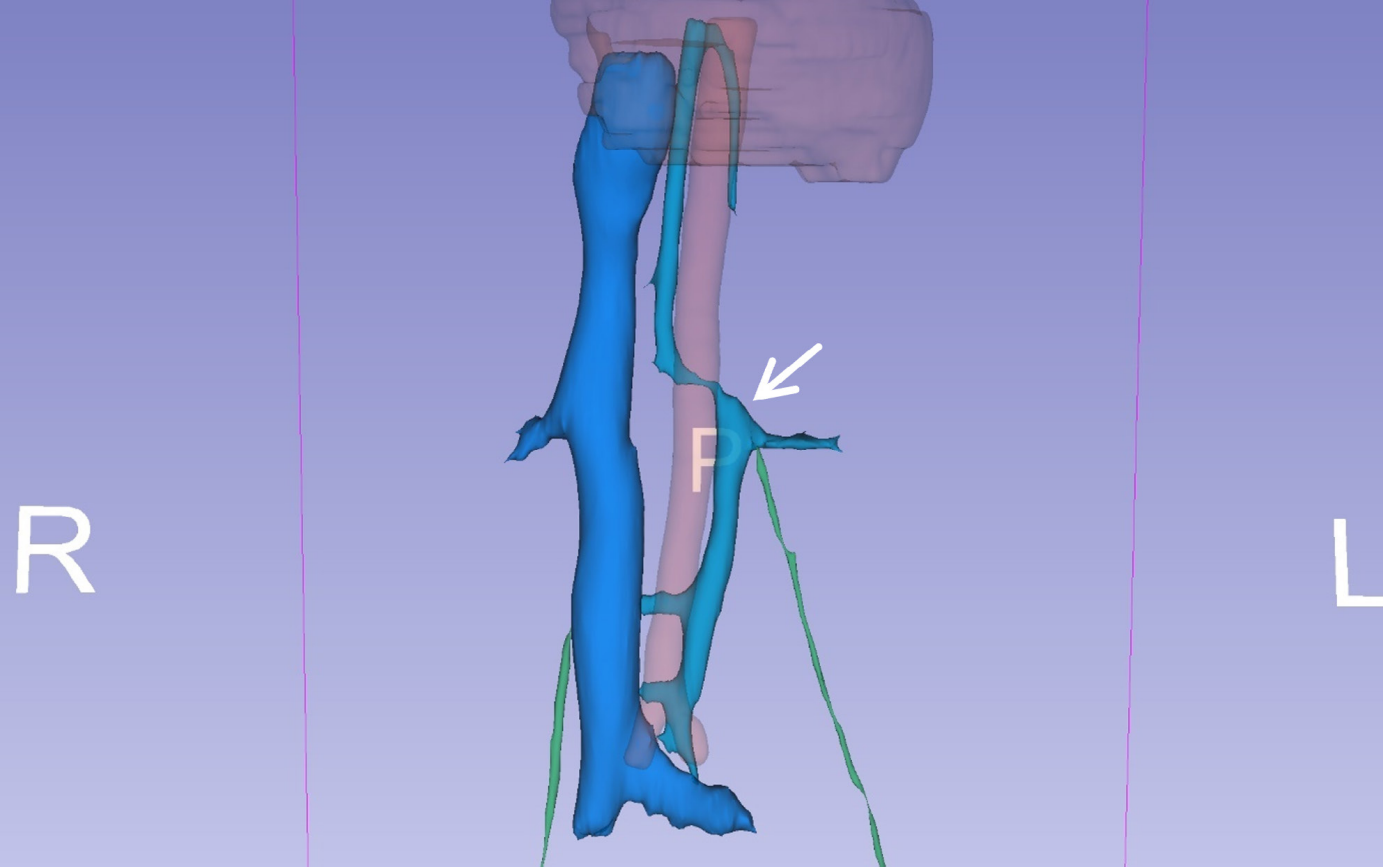
Operator assisted 3D reconstruction of main retroperitoneal vasculature with 3D slicer application version 5.0.2. Aorta (red); right renal vein, right IVC and common iliac veins (dark blue); azygos vein, accessory hemiazygos vein, communication between azygos and left IVC, left renal vein, left IVC, its three visible communications to the right IVC and joint of the left IVC with left common iliac vein (cyan); right and left gonadal veins, with the later joining distal of the left renal vein (green). Right (R), left (L) and posterior (P) sides of the image are labeled. superior segment of the left IVC (arrow) above the confluence of the left renal vein is marked. Note the mostly vertical course of the left IVC and its perpendicular angle to left renal vein.

In a more in‐depth review of the CT by the radiology department, the LRV was observed to be draining into a venous structure (diameter 9.7 mm) positioned on the left side of the abdominal aorta parallel to it and the IVC and perpendicular to the LRV (Figure 1, Figure 2, Figure 3). This structure itself had several connections to the IVC—at L4 and L5 levels—and the left common iliac vein (CIV) caudally. Furthermore, it was cranially connected to the azygos vein (diameter 7.2 mm) by crossing the midline while being compressed between the L1 vertebrae and aorta. The slightly prominent left gonadal vein drained into the LRV immediately before the LRV confluence in a normal pattern. In addition, several vertebral veins at levels of L2 to L4 vertebrae drained into this venous structure. IVC on the right side of the aorta was seen forming from the confluence of the two CIVs and draining into the right atrium after receiving the right renal vein and hepatic tributaries at normal positions.

These findings are shown at axial planes in Figure 1A–G and coronal planes in Figure 2A,B.

Since the caliber of the mentioned structure was larger than its continuation to the azygos vein and the left gonadal vein was also prominent, we asked the patient to return for an additional ultrasound study.

No other anatomical and congenital anomaly was observed in the abdominopelvic CT, especially in the genitourinary system.

## 3 METHODS

### 3.1 Investigations

**FIGURE 4:**
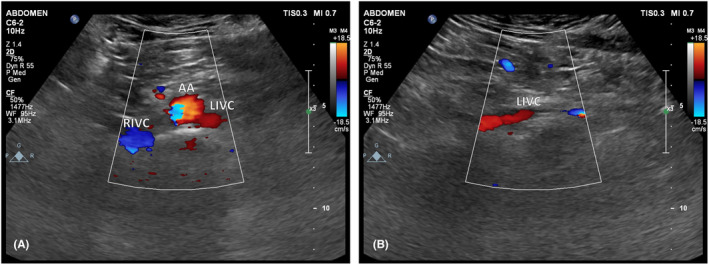
Doppler ultrasound study of the abdomen. (A) Transverse plane through the abdomen at an infrarenal level showed three retroperitoneal vascular structures. Right IVC (RIVC), abdominal aorta (AA), and left IVC (LIVC) are demonstrated. Left IVC shows the same flow direction as the abdominal aorta. (B) Longitudinal plane through the left IVC (LIVC) again, showing craniocaudal flow direction.

**FIGURE 5:**
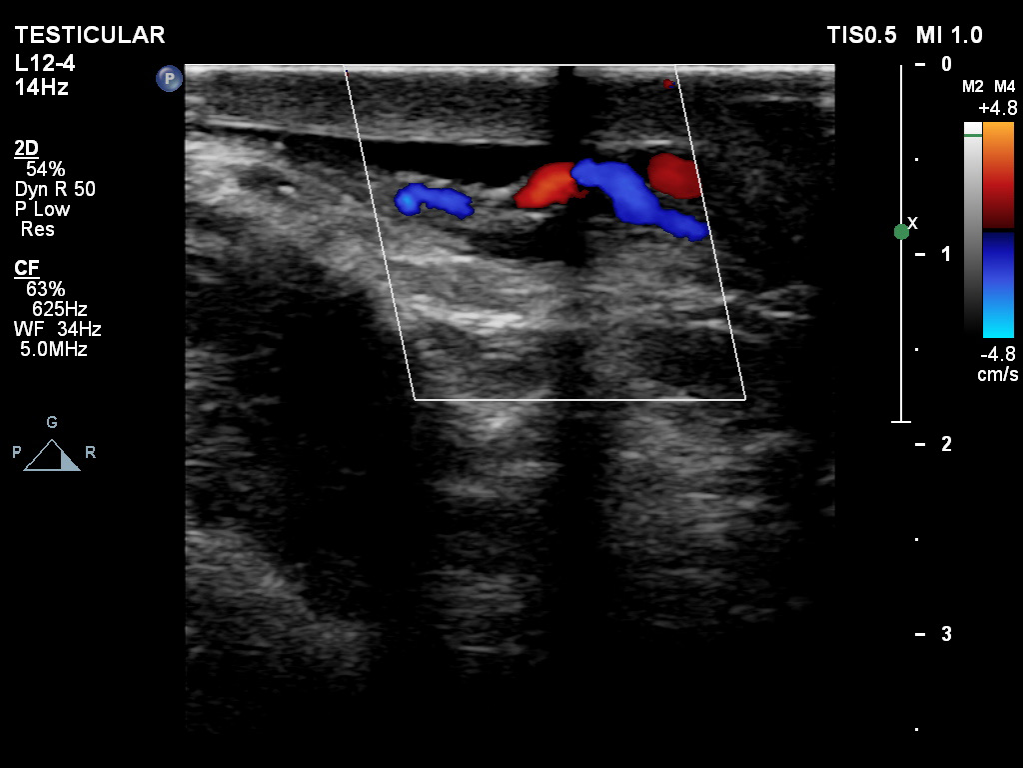
Scrotal Doppler study demonstrating enlarged left pampiniform plexus veins compatible with varicocele.

A scrotal ultrasound revealed a varicocele on the left side. On abdominal ultrasound, the vessel parallel to the IVC and aorta showed venous flow directed caudally toward the left CIV (Figures 4A, B and 5).

Although the patient had an undocumented history of left‐side varicocele, there were no other significant medical issues in his past. Specifically, there was no record of hematuria or flank pain that could be linked to nutcracker syndrome. The patient's clinical and laboratory data showed no significant findings related to the detected anatomical variation. The renal function test (creatinine level) and the estimated glomerular filtration rate (eGFR) were within normal ranges. The urinalysis performed on the day of the primary emergency room admission showed the presence of red blood cells (RBC) in the urine, which was attributed to the existing ureteral stone. The patient was treated for the ureter stone and was referred to an urologist for his varicocele.

### 3.2 Differential diagnosis

A list of differential diagnoses is mentioned in Table 1.

**TABLE 1:** Differential diagnoses of two venous structures alongside the abdominal aorta.

Anatomical variation	Major imaging features
Double IVC	IVCs on each side are an anatomic continuation of the CIV on the same side. The CIVs usually do not join each other. Although rare, the complete confluence of CIVs has been described. After receiving the Left Renal Vein, the Left IVC usually crosses the midline to join the Right IVC. Azygos and Hemiazygos continuations of the IVCs have also been reported. The Left IVC is parallel to the aorta and the Right IVC and has a perpendicular angle to the Left Renal Vein.
Retroaortic left renal vein (Type II Or IV)^a^	Usually receives the left gonadal vein before draining into the IVC at the level of L4 or L5 vertebrae (Type II) or the Left Common Iliac Vein (Type IV). Has a more oblique orientation compared to left IVC. Connections to the CIVs confluence have been reported.
Dilated left ascending lumbar vein	Positioned posterior to the psoas muscles and just anterior to transverse processes on each side of the vertebrae. Continues as the hemiazygos vein.

Abbreviations: CIV, common iliac vein; IVC, inferior vena cava; RLRV, retroaortic left renal vein.

^a^ Other types of RLRV have been proposed.^6^

On the face of it, an inexperienced eye could mistake this venous structure as part of the azygos system, that is, a dilated left ascending lumbar vein that continues as the hemiazygos vein, which itself drains into the azygos vein. However, it should be kept in mind that: (1) ascending lumbar veins are positioned posterior to the psoas muscles and take a course just anterior to transverse processes on each side of the vertebrae; (2) hemiazygos vein mostly either continues to drain to the accessory hemiazygos vein or crosses the midline to join the azygos vein on the right side, at the T8 level.^7^ In our case, the mentioned venous structure was not positioned in the expected anatomical location of the ascending lumbar vein and the hemiazygos vein could be seen separately connecting to the azygos vein at its anticipated level, thus dismissing it as being part of the azygos system.

Our main dilemma was whether to consider this venous structure as a double IVC or a retro‐aortic LRV (RLRV) since the color‐doppler ultrasound study revealed caudal venous flow rather than a cranial flow expected from a normal IVC. However, the orientation of the mentioned structure resembled an IVC rather than a type IV RLRV (explained in the discussion). Ultimately, we decided to identify the debated venous structure as a bizarre case of a double IVC—which cranially participates in the formation of the azygos vein by a narrow connection and caudally joins the left CIV and the right IVC—with a reversed flow due to the small caliber of the azygos connection.

In the end, we decided not to perform a contrast‐enhanced CT (CECT) study. The combination of a non‐contrast‐enhanced CT (NCECT) and Doppler ultrasound study provided sufficient anatomical information. A CECT would not necessarily reveal more useful information for this patient's care despite exposing him to the potential adverse effects of radiation and contrast agents.

## 4 DISCUSSION

Regression or persistence of different segments of three complex paired venous networks—known as subcardinal, supracardinal, and postcardinal systems—in the fourth to eighth weeks of embryonic life determines the final morphology of the caval and azygos veins and their tributaries. Notably, these embryonic venous channels do not necessarily exist simultaneously.^4^

The renal segment of the IVC is formed by the right suprasubcardinal and postsubcardinal anastomoses. The infrarenal segment of the IVC drives from the right supracardinal vein. Duplication of the IVC occurs due to the persistence of both supracardinal veins, instead of just on the right side.^1^, ^4^ A circum‐aortic venous collar is formed by communications between subcardinal and supracardinal channels, encircling the aorta. The ventral portion of this collar persists and becomes the normal LRV. If the dorsal portion fails to regress, it will create a retro‐aortic LRV.^8^ In the thoracic region, the caudal segments of supracardinal veins form the azygos and hemiazygos veins, and in the pelvis, the CIVs are driven from the postcardinal veins.^1^

Duplicated IVC is estimated to have a prevalence of 0.3%–4%.^4^, ^5^, ^9^ In a study, it was suggested that interiliac connections exist in two‐thirds of cases; however, the complete confluence of CIVs is rare.^5^, ^10^, ^11^ Pineda et al. have described a case of IVC duplication, which originated from the confluence of the CIVs and were reunited at the level of the renal veins. Both IVCs had approximately equal caliber.^12^ In an article published by Polguj et al., they reported another case of a double IVC originating from the confluence of the CIVs but with a smaller caliber of the left IVC. They reported that the caliber of the left IVC was significantly lower than that of the right IVC. Besides, instead of the reunion of IVC trunks at the level of the renal veins, the duplicated left IVC continued as the hemiazygos vein after it was joined by the LRV. However, the authors did not specify the flow direction in the left IVC.^5^

The incidence of the RLRV has been reported to be up to 3.6% in some studies.^13^ Four types of RLRVs are described in the literature regarding their drainage sites: (I) orthotopic drainage to the IVC; (II) draining to the IVC at L4 and L5 levels; (III) circum‐aortic LRVs; (IV) draining caudally to the left CIV via an oblique course.^8^ The most and least common types are types I and IV, respectively.^14^ The main differential diagnosis in these cases is IVC duplication. Some authors have suggested that CIVs do not fuse in typical congenital double IVC and left IVC ends at the level of the normal LRV and crosses anterior to the aorta to join right IVC.^8^ But as mentioned, rare cases of duplicated IVC that do not follow these rules have been reported.^5^, ^12^, ^15^ Sonawane et al. reported two cases of RLRVs and proposed them as new type V and VI variants of RLRV.^6^ The first case they reported had a perpendicular posture similar to our patient's LRV and vascular structure that we identified as the left IVC. It bifurcated into two segments, one draining into a normal IVC at the level of L4‐L5 and the other draining into the left CIV. No connection between the LRV and the azygos or hemiazygos veins, nor any drainage from the lumbar veins was mentioned. However, most of the other reported type IV RLRV cases show a curved oblique course toward the left CIV.^6^, ^14^, ^16^, ^17^ Although in the doppler ultrasound study, the anomalous vein that we detected showed caudally directed venous flow, we still considered it a left‐side IVC. Because the LRV was connected to it at a perpendicular angle. The mostly vertical left IVC in our patient, had two other connections with the right IVC before it joined the left CIV; unlike a true RLRV type IV that is expected to have a curved oblique course toward the left CIV. Also, it had an 8 mm cranial segment above the confluence of the LRV before crossing the midline and participating in the azygos vein formation at the T12 level (Figure 1B; 2A; 3). These differences highlight the various venous structures during embryological development of the IVC.^1^

Historically, the azygos vein and its counterpart on the left side—the hemiazygos vein—are considered to be formed by the union of the ascending lumbar and subcostal veins on each side. But contributions of IVC and LRV to the azygos vein formation at the level of T12 have also been described.^18^, ^19^ Alves et al. studied the origins of the azygos vein formation in 30 cadavers and reported that LRV participated in 6.66% of cases.^19^ Hemiazygos continuation of left IVC has also been described.^1^, ^4^ But to our knowledge, azygos continuation of left IVC has not been reported in the literature.

Both double IVC and RLRV variations can result in several complications, such as posterior nutcracker syndrome (entrapment of LRV between the abdominal aorta and vertebral body), varicocele, and recurrent pulmonary embolism following the implantation of IVC filter. It has been suggested that duplication of IVC may also increase the incidence of thrombosis formation.^1^ Additionally, the presence of double IVC or RLRV may complicate urologic and abdominal vascular surgeries.^20^ Our patient did not seem to have any debilitating conditions regarding this anomaly, except for probable flow reflux in the left testicular vein and corresponding left‐side varicocele, which we believe is a result of the discussed anatomical variation.

## 5 CONCLUSION

Although the variations and anomalies of the IVC and its correlating venous structure are thoroughly described in the literature, extremely rare and previously unreported variations should still be expected based on their embryological origins.

The lack of contrast‐enhance images may be the main limitation of this case. However, despite being a non‐vascular and non‐contrast‐enhanced study a routine abdominopelvic CT scan revealed valuable information regarding the anatomy of the main venous trunks and the anomalous structure. The retroperitoneal fat helps to distinguish the major vascular structures from the surrounding soft tissues, resulting in adequate resolution. A thorough examination of vascular structures in an NCECT should not be neglected, even though a contrast‐enhanced study could potentially reveal more details about minute and less significant connections of the IVC and azygos systems.

## AUTHOR CONTRIBUTIONS

**Adeleh Dadkhah:** Conceptualization; investigation; supervision; writing – review and editing. **Saeed Akbarzadeh Pasha:** Conceptualization; investigation; writing – original draft; writing – review and editing. **Mohammad Amin Borjian:** Investigation; writing – review and editing.

## FUNDING INFORMATION

All expenses of the current study have been covered by the authors. No external funding source was used.

## CONFLICT OF INTEREST STATEMENT

The authors declare that they have no competing interests.

## ETHICS STATEMENT

Written informed consent was obtained from the patient to use and publish his anonymized medical records. Anonymous use of the patient's data was approved by the ethics committee of Hasheminejad Kidney Center.

## CONSENT

Written informed consent was obtained from the patient to publish this report in accordance with the journal's patient consent policy.

## Data Availability

The dataset generated and/or analyzed during the current study is available from corresponding author on reasonable request.
